# Supplementary figures and images for: Up-Regulation of Astrocytic Fgfr4 Expression in Adult Mice after Spinal Cord Injury
Source: Cells. 2023 Feb 6;12(4):528. doi: 10.3390/cells12040528 (PMC9954417; doi:10.3390/cells12040528)

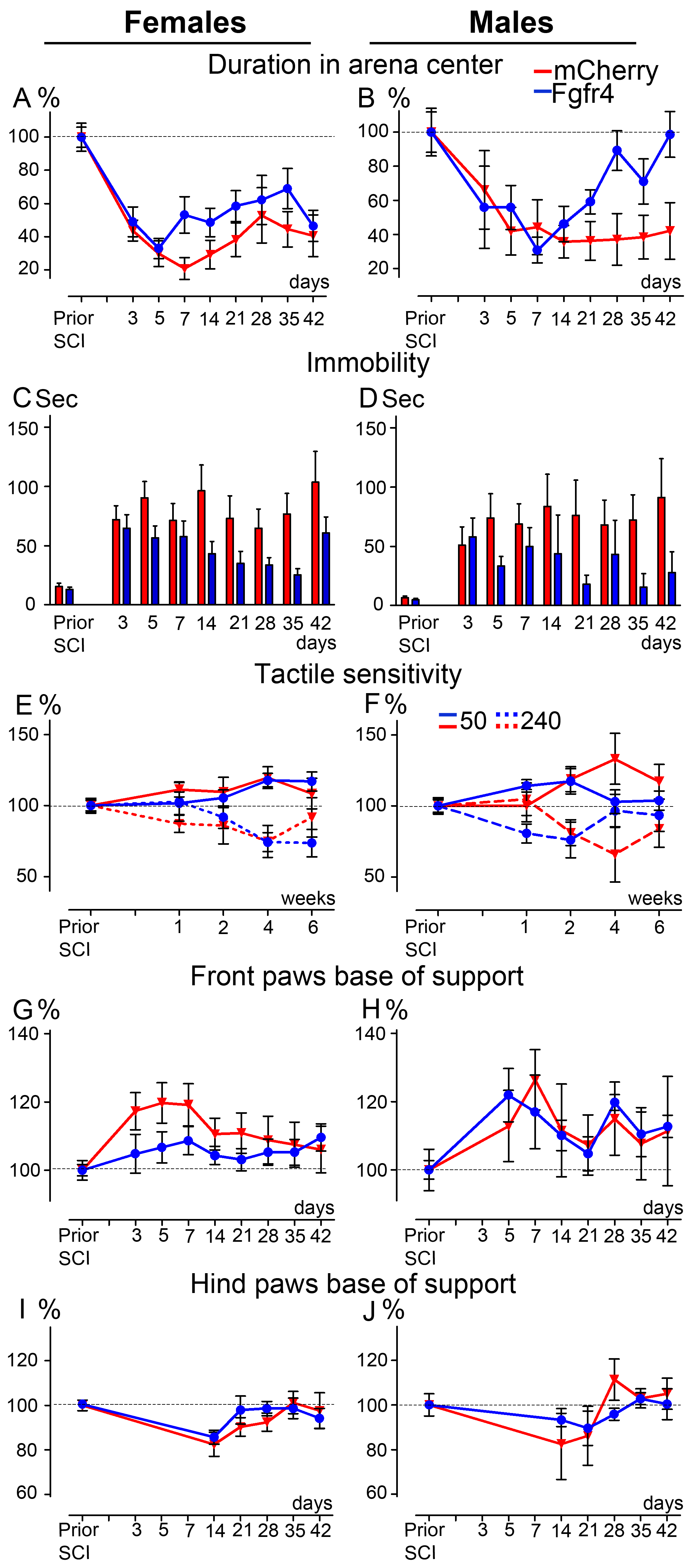

Supplement: Supplementary file 1 [file cells-12-00528-s001.zip › Bringuier et al. Figure S1.tif]

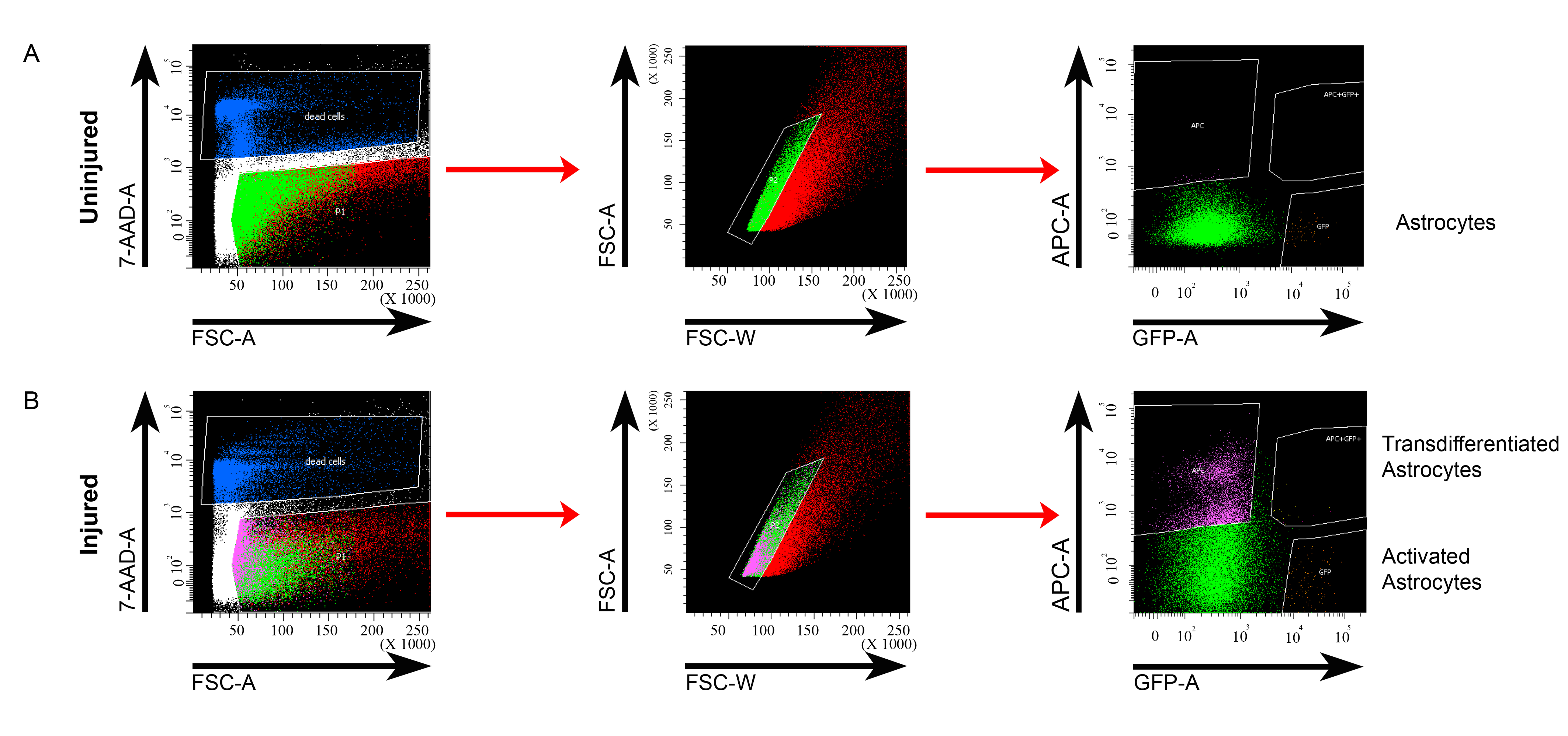

Supplement: Supplementary file 1 [file cells-12-00528-s001.zip › Bringuier et al. Figure S2.tif]
